# Sepsis survivors monitoring and coordination in outpatient health care (SMOOTH): study protocol for a randomized controlled trial

**DOI:** 10.1186/1745-6215-15-283

**Published:** 2014-07-11

**Authors:** Konrad Schmidt, Paul Thiel, Friederike Mueller, Katja Schmuecker, Susanne Worrack, Juliane Mehlhorn, Christoph Engel, Katja Brenk-Franz, Stephan Kausche, Ursula Jakobi, Anne Bindara-Klippel, Nico Schneider, Antje Freytag, Dimitry Davydow, Michel Wensing, Frank Martin Brunkhorst, Jochen Gensichen

**Affiliations:** 1 Institute of General Practice and Family Medicine, Jena University Hospital, Bachstrasse 18, 07743 Jena, Germany; 2 Institute for Medical Informatics, Statistics and Epidemiology, University of Leipzig, Härtelstrasse 16-18, 04107 Leipzig, Germany; 3 Center of Clinical Studies, Department of Anaesthesiology and Intensive Care Medicine, Jena University Hospital, Salvador-Allende-Platz 27, 07747 Jena, Germany; 4 Radboud University Nijmegen Medical Centre, Geert Grooteplein 9, PO Box 9101 6500, HB Nijmegen, Netherlands; 5 Center of Sepsis Control and Care (CSCC), Jena University Hospital, Erlanger Allee 101, 07747 Jena, Germany; 6 Department of Psychiatry and Behavioral Sciences, University of Washington, School of Medicine, 325 Ninth Ave, WA 98104 Seattle, USA

**Keywords:** Severe sepsis, Sequelae, Critical illness, Primary health care, Aftercare

## Abstract

**Background:**

Sepsis sequelae include critical illness polyneuropathy, myopathy, wasting, neurocognitive deficits, post-traumatic stress disorder, depression and chronic pain. Little is known howlong-term sequelae following hospital discharge are treated. The aim of our study is to determine the effect of a primary care-based, long-term program on health-related quality of life in sepsis survivors.

**Methods/Design:**

In a two-armed randomized multicenter interventional study, patients after sepsis (n = 290) will be assessed at 6, 12 and 24 months. Patients are eligible if severe sepsis or septic shock (ICD-10), at least two criteria of systemic inflammatory response syndrome (SIRS), at least one organ dysfunction and sufficient cognitive capacity are present. The intervention comprises 1) discharge management, 2) training of general practitioners and patients in evidence-based care for sepsis sequelae and 3) telephone monitoring of patients. At six months, we expect an improved primary outcome (health-related quality of life/SF-36) and improved secondary outcomes such as costs, mortality, clinical-, psycho-social- and process-of-care measures in the intervention group compared to the control group.

**Discussion:**

This study evaluates a primary care-based, long-term program for patients after severe sepsis. Study results may add evidence for improved sepsis care management. General practitioners may contribute efficiently to sepsis aftercare.

**Trial registration:**

U1111-1119-6345. DRKS00000741, CCT-NAPN-20875 (25 February 2011).

## Background

Sepsis is a worldwide major health concern with increasing incidence [1]. About 85,000 patients a year survive severe sepsis or septic shock [2,3] in Germany. Main sepsis sequelae include critical illness polyneuropathy/myopathy, cognitive deficits and chronic pain, all symptoms of neuronal degeneration [4-7]. In addition, post-traumatic stress disorder (PTSD) and depression are prevalent after stress exposure in the intensive care unit (ICU) [8,9].

Thus, the majority of sepsis survivors suffer from a considerable deficit in physical and psycho-social functions [10], showing a reduced health-related quality of life [11-13]. In addition to the individual burden, sepsis causes significant health economic costs, about four to seven billion Euro/year for Germany alone, including indirect costs due to loss-of-work [3].

At least, sepsis sequelae are considered to be generic for symptoms after critical illness in general [13], which is of even more clinical and socioeconomic relevance.

To improve care for long-term conditions, coordination of the fragmented process by structured interventions is effective [14]. The British National Institute for Health and Clinical Excellence’s guideline *Rehabilitation after critical illness* states that *‘*evidence is often missing and only incorporates aftercare for up to three months’ [15]. Based on a current systematic review, few follow-up interventions have been published [16]. Of these, Hacking *et al*. observed functional improvement of amputations associated with sepsis by an intensive rehabilitation program [17]. Jones *et al*. presented an increase of physical function in 126 critical illness patients who were provided a self-help manual for six months [18]. A British pilot study of Jackson *et al*., with 21 critical care patients, showed a non-significant reduction of post-traumatic symptoms using an in-house, multifaceted telemedicine program [19]. In contrast, a home-based rehabilitation program for eight weeks did not lead to better health-related quality of life or to better physical function in Australian critical illness patients [20]. A nurse-based translational pilot program that incorporated case management for patients with chronic critical illness was not effective for clinical or psycho-social outcomes [21,22].

After ICU care and hospital-based rehabilitation, most sepsis survivors receive aftercare from their primary care physician, as with most chronically ill patients. This setting is characterized by a long-lasting doctor-patient relationship, with all health services being coordinated [23]. Primary care-based interventions to improve sepsis sequelae are still rare [16].

This study will evaluate the effects of a primary care-based intervention to improve aftercare for sepsis survivors.

## Methods/Design

### Aim of the study

This study will evaluate whether health-related quality of life (SF-36) and further clinical, psycho-social, process-of-care outcomes and costs of sepsis survivors will be improved by a primary care-based, sepsis-specific aftercare program.

### Scientific hypothesis

After six months, the intervention group will show an improved primary outcome (SF-36) compared to patients with usual care in the control group.

### Study design

The study is a multicenter, prospective, two-armed randomized controlled trial. Since the intervention could compromise educational elements for primary care physicians and patients, we are not able to perform a blinded intervention. GPs are allocated only to one patient - either to the control or intervention group.

### Sample size

A wide range of effect sizes is found in comparable studies. Jackson *et al*. [19] reported that for executive functioning ability a Cohen’s d =1.1 Elliott *et al*. [20] reported that for the SF-36 physical and mental summary scores, Cohen’s d = 0.14/0.13, respectively. For our study, we assume a medium Cohen’s d = 0.5 for our primary outcome, which is between these ‘extreme values’.

With a statistical power of 90% and a significance level of 0.05, we need n = 172 patients at T2 for two-sided tests. Assuming a 40% drop-out rate [24] and 30% mortality [25], 290 patients are needed at T1.

### Data collection

Patients will be recruited from 20 ICUs in nine study centers across Germany (see Additional file 1). Eligible survivors of severe sepsis or septic shock are screened on a daily basis by ICU consultants and reported to the study team. Within one month after discharge from ICU, patients are contacted by a study physician and asked to participate in the study. For eligibility, a cognition test is performed [26]. All patients are informed about the study course.

Within one month after discharge from ICU, the first data set (T1) is collected by the study nurse, including clinical and socio-demographic characteristics. At the same time, the liaison physician contacts the responsible general practitioner (GP) by telephone to ask for study participation.

### Randomization

Given the GP’s written consent, patients are randomized in the intervention versus control group with n = 145 patients per group. Randomization sequence is computer-generated and provided in a sealed opaque envelope.

### Inclusion criteria

Patients are eligible on the presence of severe sepsis or septic shock, as defined by the definitions of the German Sepsis Society [27]. Two systemic inflammatory response syndrome (SIRS) criteria have to be completed and at least one organ dysfunction (see Additional file 2). Inclusion criteria are checked by the participating ICU doctors.

Furthermore, patients must be 18 years or older and capable of sufficient German language skills.

### Exclusion criteria

Patients are excluded from study participation due to insufficient German language skills, deafness, blindness or speech impairment. Furthermore, patients suffering from severe cognitive impairment are not eligible for study participation (determined by a telephone interview of cognitive status (TICS-M) ≤27 points) [26].

### Intervention treatment

**Figure 1:**
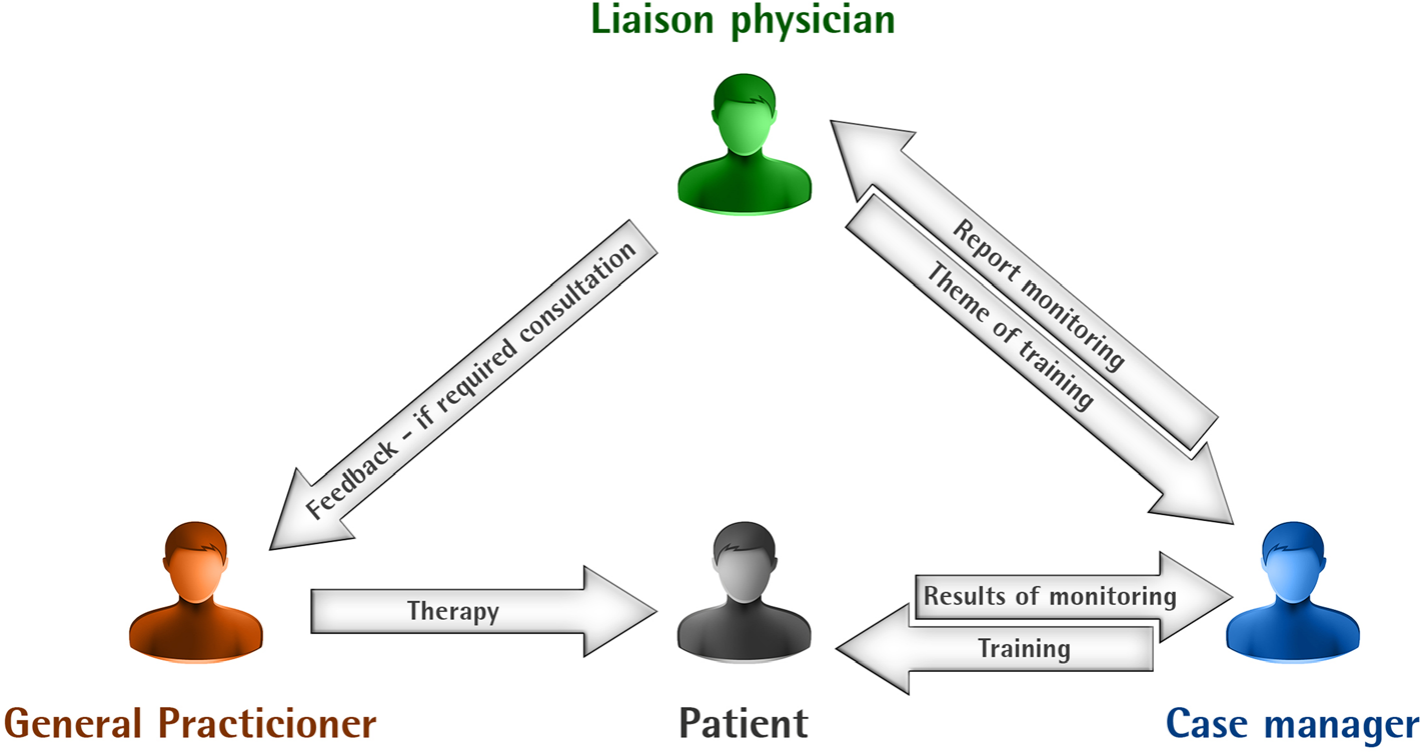
Players of the intervention.

The intervention contains three main components:

1. Discharge management with structured information between inpatient and outpatient care in accordance with the transitional care model [28], which was shown to reduce costs and rehospitalization rates [29].

2. Training of GPs and patients in sepsis sequelae and evidence-based treatment options [30]. A special focus lies on an effective self-management of patients, which improves health-related quality of life in post-intensive care patients [31].

3. Monthly telephone monitoring of patients through specifically designed telephone interviews is provided for 12 months regarding sepsis sequelae symptoms. Systematic monitoring improves the physician-patient interaction and supplies relevant information on patient clinical status to the GP, according to the chronic care model [32,33].The intervention is delivered by a study center-based case manager and a liaison physician (see Figure 1). The case manager is a nurse by qualification, trained in sepsis aftercare. She acts as an attendant for the patient, asking about the patient’s health constitution and actual problems, and provides training and monitoring. The liaison physician as the GP’s contact person is responsible for the education and reporting of the monitoring results and offers feedback if required. The liaison physician also determines further patient educational elements to the case manager.

After discharge from the ICU, intervention patients receive specific discharge forms from the case manager. All treating physicians (ICU, general wards and rehabilitation clinic) are requested to record sepsis-related clinical and social information about the patients and his or her needs. This supports the GP to manage ambulant treatment and special therapeutic needs of the patient like home care, physiotherapy, specific adjuvants *etcetera.*

Both, patients and GPs are trained in evidence-based diagnostics and therapy of the most prevalent sepsis sequelae. The liaison physician trains the GP face-to-face using audiovisual education material to impart knowledge about etiology, symptoms, diagnostic instruments and therapy options of sepsis sequelae.

Patients in the intervention group are educated as well in a face-to-face situation by the case manager to get better information about:

1. the study course,

2. the monitoring program,

3. origin and therapy of sepsis,

4. possible sepsis sequelae,

5. physical and psychological impacts of intensive therapy and

6. coping strategies and self-efficacy.

Patients and GPs receive a written manual, including both training content and monitoring instruments. Manuals are used to support training sessions. The patient manual is based on the Discern-criteria [34]. Patients are contacted every month during the first six months after discharge from ICU and every three months during months 7 to 12 for the monitoring. This telephone interview includes established short form instruments for the most common sepsis sequelae, differing from outcome instruments (see Additional file 3). Based on the monitoring results, patients are encouraged to work toward target agreements in daily life. Patient compliance is requested and monitored in the course.

The liaison physician provides this information to the GP with the help of a stratification of urgency (traffic light scheme). The GP is contacted immediately by phone if a new clinical condition arises and gets information about diagnostic and therapeutic possibilities, whereas the liaison physician is available for supervision and requests. In addition, the patient is asked to visit his/her GP if health problems occur.

### Control treatment

Patients in the control group are treated as usual by their GPs or ambulant specialists without any additional information or monitoring. There aren’t any outpatient sepsis follow-up clinics in Germany.

Due to the lack of sepsis aftercare guidelines in Germany, there is no treatment standard available. Usual care is assessed in control group patients. Qualitative interviews of GPs and patients provide additional information.

### Data collection

Data are collected from patients in questionnaires by trained study staff, initially face to face, and from T2 onwards, by phone call. In addition, GPs and ICU staff are asked to provide clinical information after patients have given their informed and written consent. Data are documented as written case report forms and stored in a protected cabinet.

### Outcome measures

Measurements take place from 3 months before hospitalization (**T-1**, retrospectively) until 1, 6, 12 and 24 months after discharge from the ICU (**T1-4**).

Primary outcome is the health-related quality of life assessed by the Short Form 36 Health Questionnaire (SF-36), a multidimensional construction of physical, mental, social and behavior-related components of well-being and operational capability, validated also for German primary care [35].

Secondary outcomes focus on the most relevant sepsis sequelae, including the assessment of physical activity, level of pain, and cognitive deficits or neuropathic symptoms by established questionnaires, all of which are patient-reported outcomes.

Furthermore, for GP compliance review, treatment and cost-effectiveness analysis, patient rehospitalizations, medication, care needs, mortality, physical therapy and instrumental diagnostic procedures are documented by the primary care provider. GPs are asked to provide details of their practice characteristics.

For clinical analysis, diagnoses and ICU procedures are extracted from the ICU documentation system.

For detailed variable/outcome parameter descriptions see Table 1 and Additional file 4.

**Table 1:** List of variables/outcome parameters

**Variables**	**Time of measurement**	**Instrument used (number of items)**
Intensive care unit (ICU)		
Documentation		
ICU stay	T_1_	Days
Mechanical ventilation	T_1_	Days
Kidney replacement therapy	T_1_	Days
Diagnoses at ICU discharge	T_1_	ICD-10
Focus of infection	T_1_	schematized (13)
Microbiological analysis	T_1_	pathogen cluster (8)
Use of sedatives, steroids	T_1_	schematized (4)
Patient reported		
Educational status	T_1_	schematized (2)
Socio-economic status	T_-1 to 4_	schematized (8)
Outcome measure (patient ratings)		
Health-related quality of life	T_-1 to 4_	Short Form 36 Health Survey (SF-36) (36) [35]
Depressive symptoms	T_1 to 4_	Major Depression Inventory (MDI) (12) [36]
Post-traumatic symptoms	T_1 to 4_	Post-Traumatic Stress Syndrome 10-Questions Inventory (PTSS-10) (10) [37]
Motoric function	T_2 to 4_	Short Musculoskeletal Function (XSMFA-D) (16) [38]
Impairment of swallowing, hearing, smelling	T-_1 to 4_	4-stepped Likert scale (4)
Chronic pain	T_1 to 4_	Graded Chronic Pain scale (GCPS) (7) [39]
Neuropathic symptoms	T_1 to 4_	Neuropathic Symptom Score (NSS) (6) [40]
Nutritional status	T-_1 to 4_	Malnutrition Universal Screening Tool (MUST) (4) [41]
Cognitive status	T_1 to 4_	Telephone Interview of Cognitive Status (TICS-M) (21) [42,43]
Sleep	T_2 to 4_	Regensburg Insomnia Scale (RIS) (15) [44]
Medication addiction	T_2 to 4_	Short form for medication use (KFM) (12) [45]
Patient assessment of care	T_-1, 2 to 4_	Patient Assessment of Care for Chronic Conditions (PACIC) (20) [46,47]
Compliance/adherence	T_-1,2 to 4_	Modified Morisky questionnaire (4) [48]
Activities of daily life	T_2 to 4_	(Instrumental) Activities of daily life (ADL/IADL) (11)
General practitioner (GP) documentation		
Mortality	T_2 to 4_	
Current diagnoses	T_-1,2 to 4_	ICD-10
GP consultation	T_2 to 4_	Number
Stay in hospital	T_2 to 4_	Days
Inability to work	T_2 to 3_	Days
Medication	T_-1, 2 to 4_	agent, dosage
Stay in rehabilitation clinic	T_1 to 4_	Days
Remedies and therapeutic aids	T_2 to 4_	schematized (1)
Nursing level	T_2 to 4_	schematized (2)
Contacts to specialists, diagnostic procedures	T_1 to 4_	schematized (7)

To gain insight into processes, barriers and mechanisms of the intervention, qualitative interviews with patients and GPs are performed on a subsample. In this context, the roles of patients’ relatives are also to be evaluated.

### Data analysis

Randomization process will be proofed using binary logistic regression. The dependent variable logarithmic odds ratio of patient is in the intervention versus control group. As predictors, we use potential confounders (for example, socio-demographic or clinical variables). Primary outcome will be analyzed based on a two-sided t-test. Secondary endpoints will be analyzed depending on scale level, using descriptive or hypothesis generating test procedures. For evaluation of average treatment effects, we will analyze the outcome variables with a generalized analysis of covariance (g-ANCOVA) and control for potential confounders. Based on these results, we are able to adjust means, which are comparable with outcome means of a perfect randomized and balanced design [49,50].

Furthermore, mortality is taken into account. Using survival analysis models, we are able to examine the relationship between mortality and treatment. In this way, we also try to identify mortality risk factors.

Finally, we plan cost-effectiveness analyses. Therefore, we sum up the costs of micro interventions and compare the averages of treatment and control group using g-ANCOVA (see above).

### Description of risks

Showing 6- month mortality of more than 30% [25], severe sepsis ranks among critical long-term conditions. Serious risks or undesired effects of completing questionnaires have not been reported by clinical expertise of the scientific experts in the advisory board of the study (see Additional file 1). There are no specific risks related to the intervention. Thus, there are no rules for stopping the intervention.

### Ethical principles

The study is planned and conducted in accordance with medical professional codex and the Helsinki Declaration of 1996 as well as the Federal Data Protection Act (BDSG).

Patients participate in the study voluntarily and give written informed consent. Patients are informed that they can cancel their participation at any time without disclosing reasons for their cancellation and without negative consequences for their future medical care. The study protocol was approved by the institutional review board of the University of Jena, 26 January 2011 (No.3001/111).

### Data security

The patient names and other confidential information are secured by the medical confidentiality rules and are treated according to Federal Data Protection Act (BDSG).

All study-related data and documents are stored on a protected central server at Jena University Hospital. Only members of the study team have access to the study files.

Intermediate and final reports are stored in the office of the Institute of General Practice and Family Medicine at the Jena University Hospital.

## Discussion

### Limitations

Contacting control patients via phone calls for data collection may create a small intervention (Hawthorne) effect in the control group. Therefore, the intervention effect is likely to be underestimated. This might be acceptable because we would not overuse any effect of the trial.

In addition, contamination by information flow between the intervention and control group cannot be excluded. However, this risk seems to be minimized by the allocation of one GP to one patient of either intervention or control group. Most GPs in Germany practice alone.

### Strengths/conclusion

To our knowledge, SMOOTH is the first study evaluating the effects of a primary care-based intervention for patients after a critical illness, that is sepsis [16]. Using established primary care structures, SMOOTH may provide a cost-effective addition to sepsis aftercare. Furthermore, considering the long-term impact of sepsis sequelae, 24-month follow-up data will be provided, which are rarely published and allow analysis of intervention sustainability. As a further innovative element, an external medical consultant in primary care (the liaison physician) might help to support quality of care in primary care settings - strengthening the GP as a reliable clinical partner for patients after critical illness.

## Trial status

The first patient was included on 28 February 2011. Patient recruitment is ongoing but not completed.

## Abbreviations

CSCC: Center of Sepsis Control and Care; g-ANCOVA: generalized analysis of covariance; GCPS: graded chronic pain scale; GP: general practitioner; GSS: German Sepsis Society; I/ADL: instrumental activities of daily life; ICD-10: international statistical classification of diseases and related health problems; ICU: intensive care unit); KFM: short form for medication use; MDI: major depression inventory; MUST: malnutrition universal screening tool; NSS: neuropathic symptom score; ODSS: modified overall disability sum score; PACIC: patient assessment of care for chronic conditions; PHQ-9: patient health questionnaire; PTSD: post-traumatic stress disorder; PTSS-10: post-traumatic stress syndrome 10-questions inventory; RIS: Regensburg insomnia scale; SF-36: short form 36 health survey; SIRS: systemic inflammatory response syndrome; SMOOTH: Sepsis Survivors Monitoring and Coordination in Outpatient Health care; STIFT: Thuringian Foundation for Technology, Innovation and Research; TICS-M: modified telephone interview for cognitive status; XSMFA-D: short musculoskeletal function.

## Competing interests

The authors declare that they have no competing interests.

## Authors’ contributions

FM, JG, KS, JM and PT participated in the study design, study conduct/data collection and in writing the final manuscript; KBF, KSc, NS and SW participated in the study design and study conduct/data collection. AF, ChE, DD, FMB and MW participated in study design and the critical revision of the manuscript. ABK, StK and UJ participated in the study conduct and data collection. All authors approved the final version of the manuscript.

## Supplementary Material

Additional file 1 Scientific advisory council and SMOOTH study centres. Click here for file

Additional file 2 SIRS/Sepsis Criteria. Click here for file

Additional file 3 Monitoring instruments. Click here for file

Additional file 4 Outcome instruments. Click here for file
