# Supplementary material for: Sepsis survivors monitoring and coordination in outpatient health care (SMOOTH): study protocol for a randomized controlled trial
Source: Trials. 2014 Jul 11;15:283. doi: 10.1186/1745-6215-15-283 (PMC4226940; doi:10.1186/1745-6215-15-283)
Supplement: Additional file 1 — Scientific advisory council and SMOOTH study centres. [file 1745-6215-15-283-S1.docx]

**Scientific advisory council**

PD Dr. med Jürgen Graf

Deutsche Lufthansa AG, Frankfurt

Prof. Dr. rer nat Ulrike Ehlert

Institute of Psychology, University of Zurich

Prof. Dr. med Günter Ollenschläger

Head of AQuMED, Berlin

Prof. Dr. med Armin Sablotzki

St. Georgs Clinics Leipzig, Dept. of Anaesthesiology, Intensive Care and Pain Management

**Study Centers**

*Berlin*

Charité University Medicine Berlin

Dr. Sybille Rademacher

Dept. of Nephrology and Medical Intensive Care

PD Didier Keh, MD, Lars Töpfer MD, Anton Goldmann, MD

Dept. of Anesthesiology and Operative Intensive Care

Vivantes Clinics Berlin

PD Siegfried Veit MD, Marcel Corea MD

Vivantes Klinikum Friedrichshain, Dept. of Anaesthesiology and Intensive care medicine

Christian Berhold MD

Friedrichshain; Dept. of Internal medicine, cardiology and conservative Intensive care medicine

Prof. Herwig Gerlach MD, Susanne Toussaint MD

Neukoelln Dept. of Anesthesiology, Operative Intensive Care and Pain Management

Lorenz Reill MD

Neukoelln, Berlin, Dept. of internal medicine- cardiology, angiology and conservative Intensive Care medicine

Margerita Metzger MD, Andrea Tobolewski MD

Hellersdorf, Dept. of Anesthesiology, Operative Intensive Care and Pain Management

Prof. Peter Lehmkuhl, MD, Joachim Mühlberg, Günter Tiedemann

Auguste Viktoria/Wenckebach, Dept. of Anesthesiology, Operative Intensive Care and Pain Management

Leila Eckholt MD

Am Urban, Dept. of Anesthesiology, Operative Intensive Care and Pain Management

Adrian Freitag, MD, Rainer Kühnemund MD

Humboldt, Dept. of Anesthesiology, Operative Intensive Care and Pain Management

Josefa Lehmke, MD

Humboldt, Dept. of Internal medicine, cardiology and conservative Intensive care medicine

PD Hartmut Kern MD, Ute Rohr MD

DRK Clinics Berlin Köpenick, Dept. of Anesthesiology, Intensive Care and Pain Management

Frank-R. Klefisch, MD

Paulinenkrankenhaus Berlin, Dept. Of Anaesthesiology and Intensive Care Medicine

*Thuringia*

PD Torsten Schreiber, MD, Andrea Geist

Zentralklinik Bad Berka, Dept. Of Anaesthesiology and Intensive Care Medicine

Prof. Konrad Reinhart, MD

Jena University hospital, Dept. of Anaesthesiology

Prof. Andreas Meier-Hellmann MD

Helios Clinics Erfurt, Dept. of Anaesthesiology and Intensive Care Medicine

*Saxonia*

Prof. Armin Sablotzki, MD

St. Georgs Clinics Leipzig, Dept. of Anaesthesiology, Intensive Care and Pain Management

Prof. Marcus Pohl, Prof. Frank Oehmichen

Bavaria Clinic for Neurological Rehabilitation Kreischa
