# Supplementary material for: Sepsis survivors monitoring and coordination in outpatient health care (SMOOTH): study protocol for a randomized controlled trial
Source: Trials. 2014 Jul 11;15:283. doi: 10.1186/1745-6215-15-283 (PMC4226940; doi:10.1186/1745-6215-15-283)
Supplement: Additional file 2 — SIRS/Sepsis Criteria. [file 1745-6215-15-283-S2.docx]

**SIRS-Criteria**

| - **Hypo-(≤36°C)oder Hyperthermia (≥38°C)** |
| --- |
| - **Tachycardia (≥90/min)** not valued when beta blocker or pacemaker therapy |
| - **Tachypnea/ Hypocapnia/ artificial ventilation** Tachypnea (≥20/min) and/ or arterial paCO_2_ ≤ 4,3 kPa (32mmHg) and / or artificial ventilation |
| - **Leucocytosis /Leucopenia/Left shift** Leukocytosis ≥ 12000/ µl or Leukopenia ≤ 4000/ µl and/ or Left shift ≥ 10% |

**Organ dysfunction**

| - **Acute Enzephalopathia** reduced vigilance, restlessness, disorientation, delirium, without influence of psychotropic pharmaceuticals |
| --- |
| - **Thrombocytopenia** Thrombocytes ≤ 100.000/µl or platelet decrease > 30% in 24h without bleeding |
| - **Arterial Hypoxia** paO_2_ < 10kPa (75mmHg) under room air, paO_2_ /FiO_2_ ≤ 33kPa (250mmHg) without manifestation of pulmonary or cardiac disease; paO_2_ /FiO_2_ with pneumonia lower than 27 kPa (200mmHg) |
| - **Arterial Hypotension** systolic arterial blood pressure ≤ 90mmHg or medium arterial blood pressure ≤ 70mmHg at least 1h despite adequate Volume supply without other causes of shock |
| - **Septic Shock** Systolic blood pressure ≤ 90mmHg or mean arterial blood pressure ≤ 70mmHg at least 2 hours or Vasopressors necessary to keep the systolic blood pressure ≥ 90mmHg or the mean arterial blood pressure ≥ 70mmHg. Hypotonia exists despite adequate volume supply without other causes of shock. |
| - **Renal Dysfunction** urinary excretion ≤ 0,5ml/kg/hour at least 1 hour despite adequate volume supply and / or increase of serum-creatinine ≥ 2x beyond the reference grange of the respective laboratory |
| - **Metabolic Acidosis** Base deficit ≥ 5.0 mmol/l or Plasma-Lactate-concentration ≥1,5 x beyond the reference grange of the respective laboratory |
