# Supplementary material for: Sepsis survivors monitoring and coordination in outpatient health care (SMOOTH): study protocol for a randomized controlled trial
Source: Trials. 2014 Jul 11;15:283. doi: 10.1186/1745-6215-15-283 (PMC4226940; doi:10.1186/1745-6215-15-283)
Supplement: Additional file 3 — Monitoring instruments. [file 1745-6215-15-283-S3.docx]

**Monitoring instruments**

| **Complication** | **Instrument** | **Cut-point** |
| --- | --- | --- |
| Depressive symptoms | Patient Health Questionnaire (PHQ-9) | >2 items more than half of the days |
| Posttraumatic symptoms | 7-Item Test | ≥4 |
| Motoric function | mod. Overall Disability Sum Score (ODSS) | >6 |
| Neuropathic symptoms | Pain Detect | >18 |
| Chronic pain |  | >5 |
| Impairment of deglutition, hearing, smelling | 4-step Likert scale | Each neg. change |
| Nutritional status | Malnutrition Universal Screening Tool (MUST) | 1 - Observation  2 – Therapy |
| Cognitive impairment | 6-Item-Screener | >3 mistakes |
