# Supplementary material for: Sepsis survivors monitoring and coordination in outpatient health care (SMOOTH): study protocol for a randomized controlled trial
Source: Trials. 2014 Jul 11;15:283. doi: 10.1186/1745-6215-15-283 (PMC4226940; doi:10.1186/1745-6215-15-283)
Supplement: Additional file 4 — Outcome instruments. [file 1745-6215-15-283-S4.docx]

**Outcome measures instruments**

The **Short Form 36 Health Survey** (SF 36) is validated for mental and physical diseases [[1](#_ENREF_38)] and in post ICU patients. [[2](#_ENREF_39)]. It consists of 36 items in eight dimensions of subjective felt health. This instrument evaluates information of physical function, physical role, somatic pain, general health perception, vitality, social operativeness, emotional role, and psychological well-being [[3](#_ENREF_35)]. The SF 36 is characterized by high internal consistence (Cronbachs alpha between 0.65 and 0.94) and excellent test re-test stability. The German version was translated and validated in accordance with the methods of the international quality of life assessment group [[3](#_ENREF_35)]. It is an often translated and international widespread used instrument. A difference of at least five points s on either the mental or functional subscale is defined as socially and clinically relevant [[3](#_ENREF_35)].

Signs of depression are assessed by **Major Depression Inventory** (MDI)/WHO-10. This instrument is based on both, ICD-10 and DSM-IV criteria. It consists of 10 Items, with a five point answer scale. The sensitivity of the MDI for major depression is between 0.86 and 0.92. Specificity varies between 0.82 and 0.86 [[4](#_ENREF_40)]. Patients with psychotic depression are excluded from the test. This instrument is also validated against a clinical interview conducted by a physician [[5](#_ENREF_41)].

Posttraumatic stress disorder is examined by **Posttraumatic Stress Scale 10**; the only questionnaire which is validated against clinical diagnostic [[6](#_ENREF_8),7] and therefore features a high reliability. It is based on DSM-IV criteria and is constructed for clinical diagnostic and research and was validated in the German version for a group of ARDS patients [[7](#_ENREF_42)].

Motoric function is assessed by **Extra-short Musculoskeletal Function Assessment Questionnaire** (XSMFA-D). This is an extract of the SMFA-D, validated on cox- and gonarthrosis patients, recommended by the American society of orthopaedic surgeons (AAOS) and used for different groups of patients [[8](#_ENREF_43)]. This questionnaire consists of 16 items composed of four function scales: daily activity, arm and hand function, mobility and impairment. The German version was validated on arthrosis patients [[9](#_ENREF_44)].

Pain is assessed by **Graded Chronic Pain Scale** (GCPS), a valid and reliable instrument, which was tested and well-established in primary care [[10](#_ENREF_45)]. This questionnaire comprises pain intensity, pain chronification and loss of activity in numeric items (0 means no pain; 10 means maximal pain). The first part asks for the last four weeks and the second part for the impairment of daily life activities, leisure time activity and labour activity during the last three months. The German version is validated since 2004 [[11](#_ENREF_46)].

Malnutrition is a common problem of hospitalized patients [[12](#_ENREF_47)]. The European society of Clinical nutrition and metabolism (ESPEN) recommends the **Malnutrition Universal Screening Tool** (MUST) for assessment of malnutrition in the ambulatory sector [[12](#_ENREF_47)]. It consists of five steps: the Body mass index, unintentional weight loss, acute illness with consecutive anorexia, sum of scores 1-3 to determine the overall risk of malnutrition and use of evidence based guidelines to develop a therapy concept. The MUST is easy to use and shows excellent inter-rater reliability and predictive validity [[12](#_ENREF_47)].

Neurocognitive deficits are assessed by **Telephone Interview of Cognitive Status** (TICS-M), which is very sensitive to detect smaller deficits of cognitive ability. It can be used by telephone or in a direct interview setting and is based on the dimensions: orientation, short term and long term memory, perception, concentration and calculation capability, semantic memory, speech repetition and comprehension. This instrument is validated in German language [13,14]. TICS-M shows a correlation of 0.94 in comparison to Mini-Mental State and a high test-retest reliability [[15](#_ENREF_50)].

Critical illness neuropathy and myopathy is assessed by **Neuropathy Symptom Score**. This instrument is divided in four symptom-cluster: Intensity, localization, exacerbation, improvement of symptoms. The sum score of all items is categorized in slight, moderate and severe symptoms [[16](#_ENREF_51)]. It is a screening instrument and further neurological examination is recommended with regard to the test category. It is easy to use in primary care.

The **Regensburger Insomnia scale** (RIS) is a short self-rating scale to evaluate cognitive, emotional and behaviour-related aspects of psychophysiological insomnia [[17](#_ENREF_52)]. It was validated on 218 insomnia patients and 94 control patients with the Pitsburgh Sleep quality index (PSQI) as a reference instrument. It consists of 10 items, evaluating experience of insomnia, sleep related thoughts and fears, use of sleep medication und subjective appraisal of performance during the day.

Impairment of daily living activities has a big influence on quality of life. The questionnaire used for evaluation of activities of **daily living and instrumental activities of daily living** consists of 13 items, that could be answered by “independent” and “needs help” [[18](#_ENREF_53)]. Patients are classified in the following categories. 0=no impairment; 1-3=mild impairment; >4 severe impairment [[19](#_ENREF_53)]. This questionnaire provides a basis for the GPs` recommendations of ambulant health care.

To assess the adherence of the patients the modified **Morisky Questionnaire** is used. It is constructed dichotomous, so the four items can be answered by “yes” or “no”. For our study the modified Morisky questionnaire is used, consisting of the three items:

“Did you ever forget to take your drugs?”

“If you felt better, did you sometimes stop taking your drugs?”

“If you felt worse by taking your drugs, did you stop taking your drugs?”

So the overall score is three, which shows a high compliance [[20](#_ENREF_57)].

The **Patient Assessment of Chronic Illness Care** (PACIC) is orientated on the chronic care model and as a generic instrument well established [[21](#_ENREF_58), [22](#_ENREF_59)]. It was modified and validated for German use [[22](#_ENREF_59)]. The short version was developed from diabetes mellitus type 2 patients and showed comparable psychometric properties [[23](#_ENREF_60)]. Each item is rated on a ten step scale. “0” means “never” and “10” means “always”. The questionnaire asks for the last six months and consists of the dimensions “Patient activation”, “performance and decision help”, problem solution and advice” and “follow up and coordination” [[24](#_ENREF_61)].

Swallowing, taste, hearing and smell troubles can occur in some cases after surviving a sepsis and might be the consequence of drug toxicity or intubation e.g. To assess these more rare complications a four step Likert scale is used, as it is established in the “Brief patient health questionnaire” (PHQ) [[25](#_ENREF_62)]. Patients can choose the items: “not at all”, “on some days”, “on more than half of the days”, “nearly every day”. This instrument is not validated, because these rare ear-nose-throat troubles are not precisely described in the literature yet.

The KFM Short form for medication use [26] was developed for addicted patients in Germany 1991, evaluating several patterns of substance abuse.

**References**

1. McHorney CA, Ware JE Jr, Raczek AE: The MOS 36-item short-form health survey (SF-36): II. Psychometric and clinical tests of validity in measuring physical and mental health constructs. Med Care 1993, 31:247–263.

2. Chrispin PS, Scotton H, Rogers J, Lloyd D, Ridley SA: Short Form 36 in the intensive care unit: assessment of acceptability, reliability and validity of the questionnaire. Anaesthesia 1997, 52:15–23.

3. Bullinger M: German translation and psychometric testing of the SF-36 health survey: preliminary results from the IQOLA Project. International quality of life assessment. Soc Sci Med 1995, 41:1359–1366.

4. Cuijpers P, Dekker J, Noteboom A, Smits N, Peen J: Sensitivity and specificity of the major depression inventory in outpatients. BMC Psychiatry 2007, 7:39.

5. Bech P, Rasmussen NA, Olsen LR, Noerholm V, Abildgaard W: The sensitivity and specificity of the major depression inventory, using the present state examination as the index of diagnostic validity. J Affect Disord 2001, 66:159–164.

6. Davydow DS, Gifford JM, Desai SV, Needham DM, Bienvenu OJ: Posttraumatic stress disorder in general intensive care unit survivors: a systematic review. Gen Hosp Psychiatry

7. Stoll C, Kapfhammer HP, Rothenhausler HB, Haller M, Briegel J, Schmidt M, Krauseneck T, Durst K, Schelling G: Sensitivity and specificity of a screening test to document traumatic experiences and to diagnose post-traumatic stress disorder in ARDS patients after intensive care treatment. Intensive Care Med 1999, 25:697–704.

8. Wollmerstedt N, Kirschner S, Bohm D, Faller H, Konig A: [Design and evaluation of the extra short musculoskeletal function assessment questionnaire XSMFA-D]. Z Orthop Ihre Grenzgeb 2003, 141:718–724.

9. Konig A, Kirschner S, Walther M, Bohm D, Faller H: I. Cultural adaptation, practicability and reliability evaluation of the musculoskeletal functional assessment questionnaire. Z Orthop Ihre Grenzgeb 2000, 138:295–301.

10. von Korff M, Ormel J, Keefe FJ, Dworkin SF: Grading the severity of chronic pain. Pain 1992, 50:133–149.

11. Klasen BW, Hallner D, Schaub C, Willburger R, Hasenbring M: [Validation and reliability of the German version of the chronic pain grade questionnaire in primary care bach pain patients]. Psychosoc Med 2004, **1**:Doc07.

12. Kondrup J, Allison SP, Elia M, Vellas B, Plauth M: ESPEN guidelines for nutrition screening 2002. Clin Nutr 2003, 22:415–421.

13. Loerbroks A, Amelang M, Sturmer T: Reproducibility of a telephone interview assessing cognitive function and depressive symptoms in older adults in Germany. Int J Geriatr Psychiatry 2008, 23:1098–1101.

14. Debling D, Amelang M, Hasselbach P, Sturmer T: Assessment of cognitive status in the elderly using telephone interviews. Z Gerontol Geriatr 2005, 38:360–367.

15. Brandt J, Spencer M, Folstein M: The telephone interview for cognitive status. Neuropsychiatr Neuropsych Behav Neurol 1988, 1:111–117.

16. Haslbeck M, Luft D, Neundörfer B, Stracke H, Hollenrieder V, Bierwirth R: Diabetische neuropathie. Diabetologie 2007, 2:150–156.

17. Cronlein T, Langguth B, Popp R, Lukesch H, Pieh C, Hajak G, Geisler P: Regensburg insomnia scale (RIS): a new short rating scale for the assessment of psychological symptoms and sleep in insomnia; study design: development and validation of a new short self-rating scale in a sample of 218 patients suffering from insomnia and 94 healthy controls. Health Qual Life Outcomes 2013, 11:65.

18. Fonda S, Herzog AR: HRS/AHEAD Documentation Report. Documentation Of Physical Functioning Measured In The Health And Retirement Study And The Asset And Health Dynamics Among The Oldest Old Study. Ann Arbor, MI: Survey Research Center University of Michigan; 2004.

19. Fraley RC, Waller NG, Brennan KA: An item response theory analysis of self-report measures of adult attachment. J Pers Soc Psychol 2000, 78:350–365.

20. Morisky DE, Green LW, Levine DM: Concurrent and predictive validity of a self-reported measure of medication adherence. Med Care 1986, 24:67–74.

21. Wagner EH, Austin BT, Davis C, Hindmarsh M, Schaefer J, Bonomi A: Improving chronic illness care: translating evidence into action. Health Aff (Millwood) 2001, 20:64–78.

22. Rosemann T, Laux G, Droesemeyer S, Gensichen J, Szecsenyi J: Evaluation of a culturally adapted German version of the patient assessment of chronic illness care (PACIC 5A) questionnaire in a sample of osteoarthritis patients. J Eval Clin Pract 2007, 13:806–813.

23. Gugiu PC, Coryn C, Clark R, Kuehn A: Development and evaluation of the short version of the patient assessment of chronic illness care instrument. Chronic Illn 2009, 5:268–276.

24. Glasgow RE, Wagner EH, Schaefer J, Mahoney LD, Reid RJ, Greene SM: Development and validation of the patient assessment of chronic illness care (PACIC). Med Care 2005, 43:436–444.

25. Bracken BA, Barona A: State of the art procedures for translating, validating and using psychoeducational tests in cross-cultural assessment. School Psychol Int 1991, 12:119–132.

26. Watzl H, Rist F, Höcker W, Miehle K: **Development of a questionnaire to assess prescription drug misuse in substance misusing patients,** edited by Heide M, Lieb H, *Sucht und Psychosomatik. Beiträge des 3. Heidelberger Kongresses* 1991, 123-139.
